# The lack of statistical power of subgroup analyses in meta-analyses: a cautionary note

**DOI:** 10.1017/S2045796021000664

**Published:** 2021-12-02

**Authors:** Pim Cuijpers, Jason W. Griffin, Toshi A. Furukawa

**Affiliations:** 1 Department of Clinical, Neuro and Developmental Psychology, Amsterdam Public Health research institute, Vrije Universiteit Amsterdam, Amsterdam, the Netherlands; 2 Department of Psychology, Pennsylvania State University, Pennsylvania, USA; 3 Department of Health Promotion and Human Behavior, Kyoto University Graduate School of Medicine/School of Public Health, Kyoto, Japan

## Abstract

One of the most used methods to examine sources of heterogeneity in meta-analyses is the so-called ‘subgroup analysis’. In a subgroup analysis, the included studies are divided into two or more subgroups, and it is tested whether the pooled effect sizes found in these subgroups differ significantly from each other. Subgroup analyses can be considered as a core component of most published meta-analyses. One important problem of subgroup analyses is the lack of statistical power to find significant differences between subgroups. In this paper, we explore the power problems of subgroup analyses in more detail, using ‘metapower’, a recently developed statistical package in R to examine power in meta-analyses, including subgroup analyses. We show that subgroup analyses require many more included studies in a meta-analysis than are needed for the main analyses. We work out an example of an ‘average’ meta-analysis, in which a subgroup analysis requires 3–4 times the number of studies that are needed for the main analysis to have sufficient power. This number of studies increases exponentially with decreasing effect sizes and when the studies are not evenly divided over the subgroups. Higher heterogeneity also requires increasing numbers of studies. We conclude that subgroup analyses remain an important method to examine potential sources of heterogeneity in meta-analyses, but that meta-analysts should keep in mind that power is very low for most subgroup analyses. As in any statistical evaluation, researchers should not rely on a test and *p*-value to interpret results, but should compare the confidence intervals and interpret results carefully.

Meta-analyses have become an indispensable tool to integrate large, often complex and sometimes conflicting bodies of evidence, and to translate the results of this research into treatment recommendations, guidelines and advice for policy measures (Higgins *et al*., 2021). The methods for conducting meta-analyses have been developed well in the past decades, and every year many thousands of new meta-analyses are published.

The methods of meta-analyses are not without problems, however. One important issue is related to heterogeneity, indicating the variability between studies that are included in meta-analyses (Higgins *et al*., 2021). Meta-analyses typically include studies that vary to a certain extent and the question always is whether they are comparable enough for their results to be pooled in a meta-analysis. Statistical heterogeneity refers to the variability in the intervention effects being evaluated in the different studies, and is a consequence of clinical or methodological diversity, or both, among the studies (Cuijpers, 2016). Statistical heterogeneity can be measured directly in meta-analyses and is often quantified by *I*^2^, which indicates the percentage of the variability in effect estimates that is due to heterogeneity rather than sampling error (chance; Higgins and Thompson, 2002).

When heterogeneity is found in a meta-analysis it is important to examine potential sources of this heterogeneity. One of the most used methods to examine sources of heterogeneity is the so-called ‘subgroup analysis’ (Sun *et al*., 2012, 2014). In a subgroup analysis, the included studies are divided into two or more subgroups, and it is tested whether the pooled effect sizes found in these subgroups differ significantly from each other by looking at the interaction between the subgroup and the treatment (Cuijpers, 2016). Subgroup analyses can be considered as a core component of most published meta-analyses and are recommended by the Cochrane Handbook for meta-analyses (Higgins *et al*., 2021).

Subgroup analyses are, however, associated with several important methodological problems (Sun *et al*., 2012, 2014). One important problem of subgroup analyses is that they are easily interpreted causally, while the results are in fact observational (Higgins *et al*., 2021). Participants in the interventions are not randomised to one of the subgroups, which means that these results cannot be interpreted in a causal way. If for example an intervention is found to be more effective in nursing homes than in other settings, this can indicate a real difference between settings, but not that nursing homes reduce the effect size estimates. For example, it can also be related to the age of participants or that in nursing homes more single persons live than in other settings. Especially when the number of studies is limited, the characteristics of the included studies can be highly correlated with each other and it would be difficult to reason what the actual causes of the subgroup differences are.

## Power calculations for subgroup analyses

An even more important problem of subgroup analyses, however, is the low power of such analyses (Pigott, 2020). The second author of this paper recently developed *metapower*, a statistical package in R for conducting power analyses for general meta-analyses (e.g., summary effect size) and subgroup analyses (Griffin, 2021). A shiny app makes the software easily accessible (jason-griffin.shinyapps.io/shiny_metapower). With this package, it is possible to examine the statistical power of subgroup analyses in different scenarios. A simple example can show that you need many more studies to detect subgroup differences than you would need to detect a main effect in the meta-analysis.

Suppose for example that we are conducting a meta-analysis comparing the effect of an intervention over a control condition in which each included study has 50 participants and a moderate degree of heterogeneity (i.e., *I^2^* = 50%). If the standardised mean difference (SMD) between treatment and control group after treatment is 0.5 in a random-effects meta-analysis, we would ‘only’ need six studies to have 80% power.

Now let us see what happens when we do a subgroup analysis, with the same assumptions (50 participants per study, *I^2^* of 50% and a random-effects model) and a difference between the two subgroups of 0.5 (one subgroup has an SMD of 0.1 and the other of 0.6; and studies in the meta-analysis are evenly divided over the two subgroups). For such a subgroup analysis, a total of 22 studies would be needed to have 80% power, almost four times the number of studies needed for the main analyses.

## Other scenarios

### Varying subgroup differences

The number of studies needed for subgroup analyses increases exponentially when the difference in effect sizes between the subgroups gets smaller. For example, subgroup differences of 0.3, 0.2 and 0.1 correspond to a required number of studies equal to 56, 120 and 498 respectively. For small subgroup differences (e.g., SMD = 0.1), the number of studies required is 83 times that needed for the main analysis. As a result, meta-analysts should carefully consider the statistical power of subgroup analyses (especially when the expected subgroup differences are small).

### Unbalanced subgroups

**Fig. 1.**
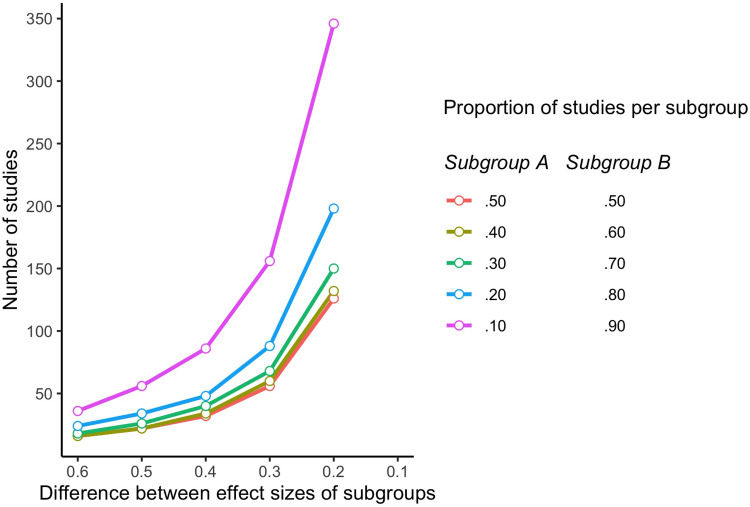
Number of studies needed in subgroup analyses within meta-analyses for 80% power, depending on the difference between the effect sizes of the subgroups and the proportion of studies in each subgroup.

Thus far, the required number of studies to achieve 80% power assumed that the studies in the meta-analysis are evenly divided over the two subgroups (50% of studies in subgroup A and the other 50% in subgroup B). What happens when the included studies are not evenly divided, but when one subgroup is larger than the other? In Fig. 1 we graphically present the number of studies needed in different scenarios for subgroup analyses. We have indicated how many studies are needed for varying differences in effect sizes between the two subgroups on the horizontal axis (ranging from an SMD of 0.6–0.1) and for the proportion of the studies in each subgroup (50% of studies in both subgroups; 40% of studies in one subgroup and 60% in the other, 30% in one subgroup and 70% in the other, etc). As shown, the number of studies increases exponentially with a decreasing difference in effect size, when each subgroup includes 50% of studies. This exponential increase is even stronger when one subgroup is larger than the other. For example, when the SMD between the two subgroups is 0.2 and one subgroup has 20% of studies (the other 80%), then more than 200 studies are needed to realise 80% power; when one subgroup only includes 10% of the studies and the other 90%, more than 350 studies are needed when the SMD between the subgroups is 0.2.

### Additional factors

Of course, the number of studies needed in subgroup analyses depends on several additional factors. When we want to use a threshold for sufficient power that is not 80% but for example 90%, the number of studies needed is also much higher. In our hypothetical meta-analysis (50 participants per study, *I*^2^ = 50%, SMD between the two subgroups of 0.5) we need 22 studies for 80% power, but 28 for 90% power. That is more than four times the number of studies needed for the main analyses on the difference between treatment and control.

Another important factor is the level of heterogeneity. When heterogeneity is lower, less studies are needed. For example, when heterogeneity is low (*I*^2^ = 25%) and the studies are evenly divided over the subgroups, 14 studies are needed for detecting an SMD between the subgroups of 0.5, more than twice the number for the main meta-analysis of our hypothetical example. When *I*^2^ = 50% we would need 21 studies. When heterogeneity is higher than 50%, considerably more studies are needed for realising sufficient power. For the field of psychology, the degree of heterogeneity has been estimated to be 74% on average (Stanley *et al*., 2018). When we assume that heterogeneity is high (*I*^2^ = 75%), then we need 42 studies in our hypothetical meta-analysis, twice the number for *I*^2^ = 50% and seven times the number to find a main effect of the intervention of a similar magnitude.

## How should subgroup analyses be used in meta-analyses?

Power calculations indicate that subgroup analyses in a meta-analysis require dozens to hundreds of studies for realising sufficient statistical power. The uncertainty of these analyses further increases when the quality of the included studies is suboptimal, and the different moderators are correlated with each other. Moreover, most meta-analyses include not one subgroup analysis for one moderator, but usually, several subgroup analyses are run for many different moderators. Subgroup analyses are also often not pre-specified, and multiple moderators may be examined while only a few are reported. Running multiple subgroup analyses can result in chance findings for only apparently significant moderators (Sun *et al*., 2012, 2014). In other words, subgroup analyses risk inflating not only beta errors but also alpha errors.

However, despite these problems with subgroup analyses it remains important to examine potential sources of heterogeneity in meta-analyses and subgroup analyses are an essential element of that. It is first of all important to keep in mind the old axiom that no evidence of a difference is not evidence of no difference. That is especially important because the power for subgroup analyses is so low. It is important to guard against alpha error inflation through limiting ourselves to a small number of subgroup analyses which should be pre-specified with reasons.

## References

[ref1] Cuijpers P (2016) Meta-analyses in Mental Health Research; A Practical Guide. Amsterdam: Vrije Universiteit. Available at http://bit.do/meta-analysis.

[ref2] Griffin JW (2021) Calculating statistical power for meta-analysis using metapower. Quantitave Methods for Psychology 17, 24–39.

[ref3] Higgins JPT and Thompson SG (2002) Quantifying heterogeneity in a meta-analysis. Statistics in Medicine 21, 1539–1558. 12111919 10.1002/sim.1186

[ref4] Higgins JPT, Thomas J, Chandler J, Cumpston M, Li T, Page MJ and Welch VA (eds) (2021) *Cochrane Handbook for Systematic Reviews of Interventions version 6.2* (updated February 2021). Cochrane, 2021. Available at www.training.cochrane.org/handbook.

[ref5] Pigott TD (2020) Power of statistical tests for subgroup analysis in meta-analysis. In Ting JCC, Ho S and Chen DG (eds), Design and Analysis of Subgroups with Biopharmaceutical Application. Springer Nature, Cham, Switzerland: Springer International Publishing, pp. 347–368.

[ref6] Stanley TD, Carter EC and Doucouliagos H (2018) What meta-analyses reveal about the replicability of psychological research. Psychological Bulletin 144, 1325–1346. 30321017 10.1037/bul0000169

[ref7] Sun X, Briel M, Busse JW, You JJ, Akl EA, Mejza F, Bala MM, Bassler D, Mertz D, Diaz-Granados N, Vandvik PO, Malaga G, Srinathan SK, Dahm P, Johnston BC, Alonso-Coello P, Hassouneh B, Walter SD, Heels-Ansdell D, Bhatnagar N, Altman DG and Guyatt GH (2012) Credibility of claims of subgroup effects in randomised controlled trials: systematic review. British Medical Journal 344, e1553. 22422832 10.1136/bmj.e1553

[ref8] Sun X, Ioannidis JP, Agoritsas T, Agoritsas T, Alba AC and Guyatt G (2014) How to use a subgroup analysis: users’ guide to the medical literature. Journal of the American Medical Association 311, 405–411. 24449319 10.1001/jama.2013.285063

